# A Brief History of Aaron T. Beck, MD, and Cognitive Behavior Therapy

**DOI:** 10.32872/cpe.6701

**Published:** 2021-06-18

**Authors:** Judith S. Beck, Sarah Fleming

**Affiliations:** 1 Beck Institute for Cognitive Behavior Therapy, Philadelphia, PA, USA; 2 Perelman School of Medicine, University of Pennsylvania, Philadelphia, PA, USA

On July 18^th^, 2021, the medical and mental health community around the world will celebrate the 100^th^ birthday of Aaron T. Beck, MD. Dr. Beck is globally recognized as the father of Cognitive Behavior Therapy (CBT) and is one of the world’s leading researchers in psychopathology. Since he developed CBT in the 1960s and 1970s, this revolutionary treatment has been found to be effective in over 2000 clinical trials for a wide range of mental disorders, psychological problems, and medical conditions with psychological components. A prolific and productive researcher with a career spanning more than 70 years, Dr. Beck has published over 600 articles and authored or co-authored 25 books. He is also the recipient of numerous awards, including the 2006 Albert Lasker Award for Clinical Medical Research and the Gustave O. Lienhard Award from the Institute of Medicine for “outstanding national achievement in improving personal health care services in the United States.” He has dedicated his life to alleviating human suffering through the development of an evidence-based psychological therapy and continues his work to this day.

CBT is based on the psychological construct that individuals’ interpretations of situations influence their reaction (emotional, behavioral, physiological), more so than the situation itself. Further, people’s interpretations may be distorted, inaccurate or unhelpful, particularly when psychopathology is present. These interpretations, termed “automatic thoughts”, are often linked to maladaptive underlying beliefs that individuals have about themselves, other people, the world, or the future. Dr. Beck found that when he helped his patients evaluate and change their distorted thinking, they felt better and were able to modify their behavior. When he helped them evaluate and change their underlying beliefs, their improvement was long-lasting.

**Figure:**
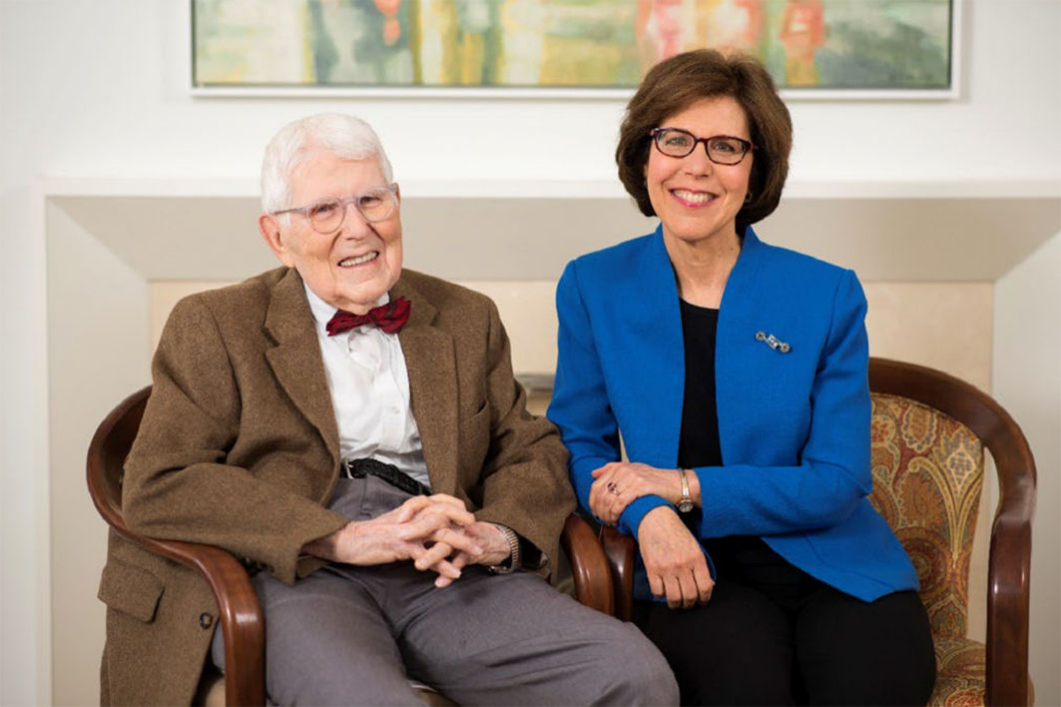
Dr. Aaron T. Beck and Dr. Judith S. Beck Co-Founded Beck Institute in 1994 Photo © 2019 Beck Institute for Cognitive Behavior Therapy

## The Development of Cognitive Therapy

As a young psychiatrist in the 1950s, Dr. Beck wholly subscribed to the dominant psychotherapeutic modality at the time: psychoanalysis. His earliest research sought to validate psychoanalytic constructs. He was surprised when his research appeared to refute the underlying tenets of psychoanalytic theory. Rather than confirm the psychoanalytic theory that depressed clients felt an innate need to suffer, Dr. Beck’s initial studies with depressed patients seemed to point to underlying negative beliefs associated with loss and failure. He soon began to understand that these underlying beliefs were consistent with the patients’ automatic thoughts, which could be accessed and collaboratively evaluated in session. Dr. Beck moved his patients from the couch to a chair, where he worked with them to examine their automatic thoughts and identify cognitive distortions. By helping patients correct negative information processing biases, he was able to help them feel better and engage in more adaptive behaviors. He called his new therapy “Cognitive Therapy”.

In 1977, the results of the first major clinical trial comparing Cognitive Therapy to anti-depressant medication were published (Rush et al., 1977). Cognitive Therapy became the first talking therapy shown to be more efficacious than medication for the treatment of depression. When a second study, conducted in the UK and published in 1981, appeared to replicate the results (Blackburn et al., 1981), interest in the approach grew nationally and internationally.

**Figure:**
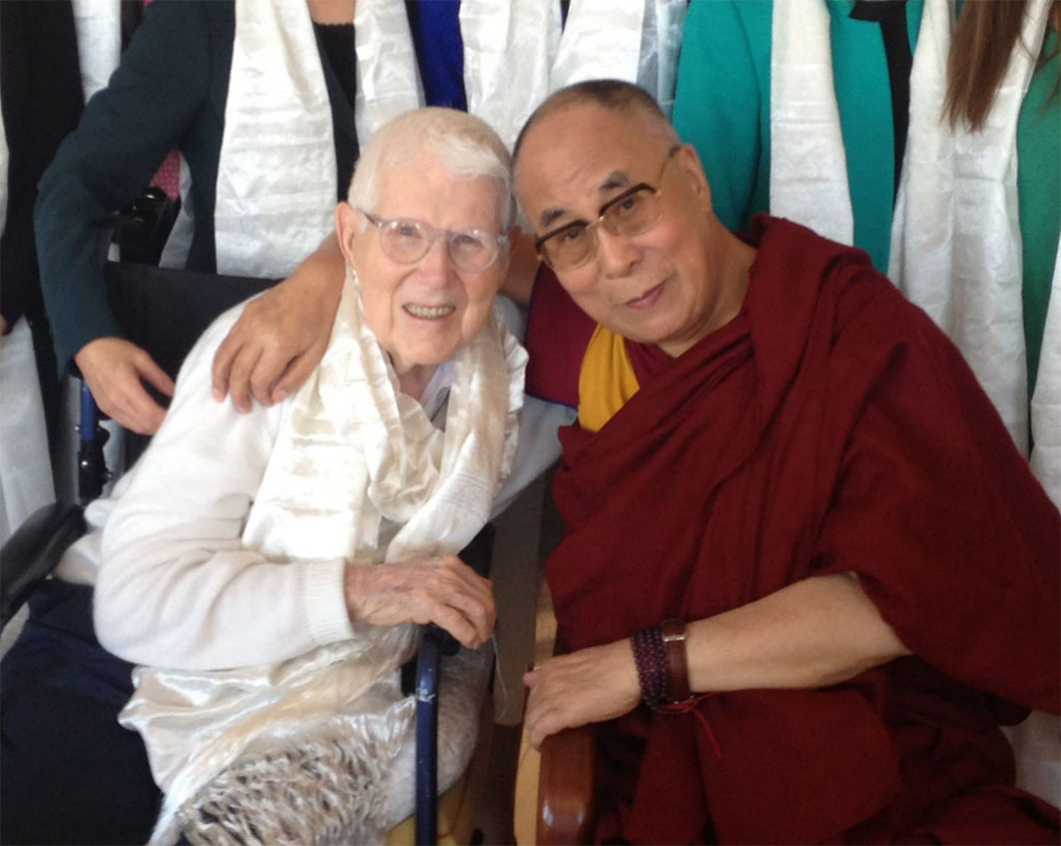
Dr. Aaron T. Beck and the Dalai Lama Photo © 2019 Beck Institute for Cognitive Behavior Therapy

Dr. Beck (and colleagues) began to apply Cognitive Therapy to other disorders, such as anxiety, personality disorders, substance use, and suicidality. He developed a comprehensive theory of psychopathology that provided the basis for treatment and methods to evaluate the validity of his theories and the efficacy and effectiveness of the therapy. For each new condition, he would begin by making clinical observations, identifying typical maladaptive beliefs associated with the disorder. He often developed scales and instruments to assess these beliefs. He would then develop a treatment to target the dysfunctional beliefs and associated maladaptive behavioral strategies. The therapy would be validated using a randomized controlled trial, then disseminated in the literature so that others could study, practice, and refine the treatment (Beck, 2019). Other researchers followed suit. In the UK, for example, a group at Oxford used a similar method to devise and test Cognitive Therapy treatment protocols for panic disorder, social anxiety disorder, obsessive-compulsive disorder, and posttraumatic stress disorder (Clark, 1986; Clark & Wells, 1995; Salkovskis, 1999; Ehlers et al., 2005). Cognitive Therapy was also successfully applied to eating disorders, couples’ problems, anger and hostility, psychosis, and other mental health problems. It was also successfully applied to children, adolescents, adults, and older adults in a variety of settings, including hospitals, outpatient clinics, residential placements, schools, and prisons.

**Figure:**
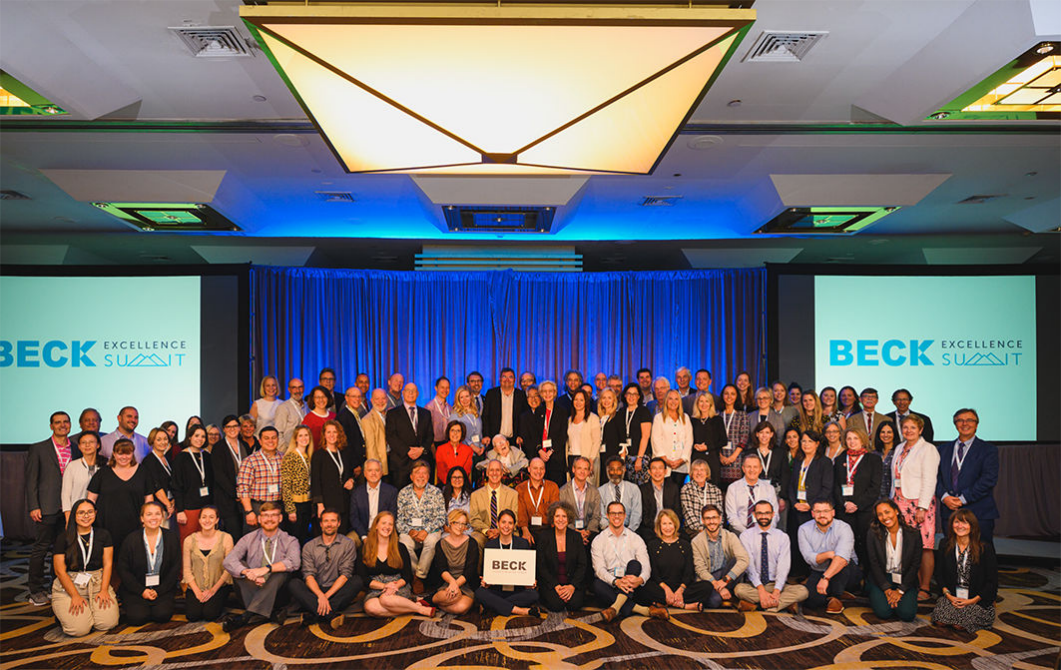
Dr. Aaron T. Beck at the Beck Excellence Summit Photo © 2019 Beck Institute for Cognitive Behavior Therapy

Additionally, researchers found that patients with medical conditions can benefit from Cognitive Therapy, or Cognitive Behavior Therapy (CBT), as it is known today. In many cases, CBT can help reduce symptoms. In other cases, CBT can help patients cope better with their conditions. Research has shown that patients with scores of medical problems from dementia and insomnia to irritable bowel syndrome, migraine headaches, obesity, and chronic pain have benefited from CBT.

## Achievements in Cognitive Therapy

CBT has become the most widely practiced (Knapp et al., 2015) and heavily researched (David et al., 2018) psychotherapy in the world. Much of its success can be attributed to the careful attention paid to its dissemination and implementation and to the training and credentialing of CBT therapists around the world. To this end, Dr. Aaron Beck and his daughter, Dr. Judith Beck, founded the nonprofit Beck Institute for Cognitive Behavior Therapy (BI) in 1994. The mission of BI is to improve lives worldwide through excellence and innovation in CBT training, practice, and research. The organization has trained more than 28,000 health and mental health professionals from 130 countries through a variety of in person and virtual programs and distance supervision, including some of the leading researchers in CBT today. All of the organization’s programs operate in service of its mission.

One of the largest and most successful implementations of CBT has been the Improving Access to Psychological Therapies (IAPT) program. Dr. David M. Clark, a prominent CBT researcher, who had maintained a close working relationship with Dr. Aaron Beck since he was a doctoral student, partnered with economist Lord Richard Layard to radically expand access to evidence-based psychological therapies throughout England via a massive overhaul of England’s National Health Service (NHS). Through IAPT, Dr. Clark and his colleagues have trained over 10,500 clinicians in CBT and other evidence-based therapies. As of 2019, one million people pass through the program each year, with over half a million receiving a course of treatment. The program has collected outcome data on 99% of those treated. Around seven in every ten treated individuals (67%) show substantial reductions in their anxiety or depression. For five in every ten (51%) the reductions are large enough for the person to be classified as recovered (Clark, 2019). By 2024, the IAPT program plans to increase its reach from one million to 1.9 million individuals annually. The IAPT program has shown that improving public mental health is not only possible but is also cost-effective. The program should serve as a blueprint for countries around the world who want to address the growing global mental health crisis.

**Figure:**
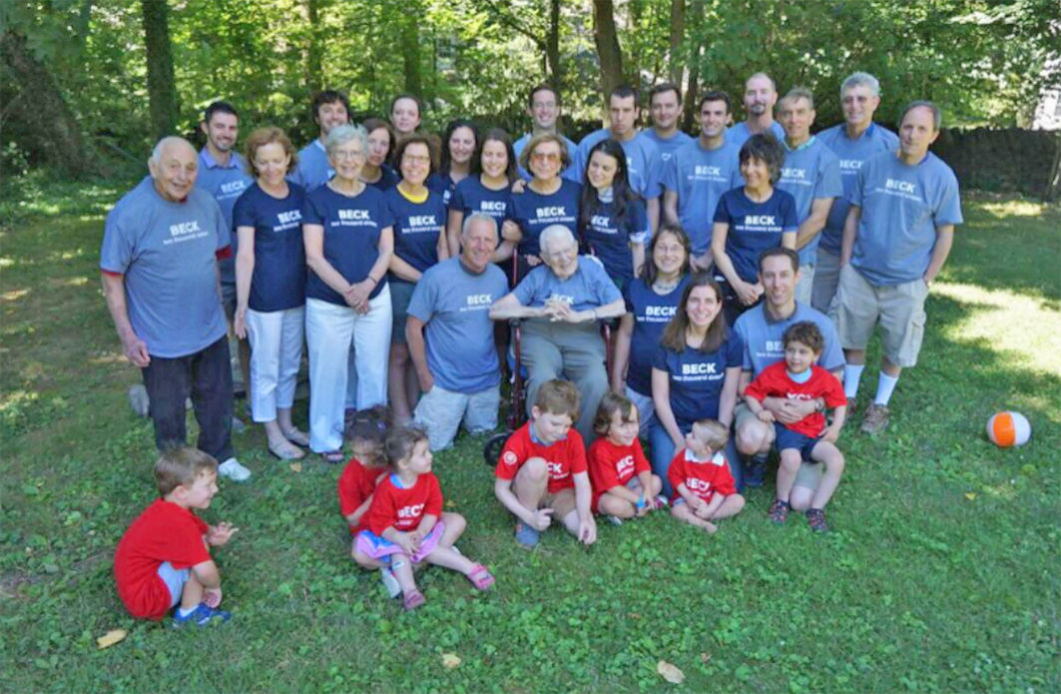
Dr. Aaron T. Beck and Family at his 95th Birthday Celebration Photo © 2019 Beck Institute for Cognitive Behavior Therapy

Dr. Aaron Beck has continued his research into the treatment of psychopathology even until today. He is most passionate about the work he and colleagues at the University of Pennsylvania and now at the Beck Institute undertook two decades ago. They developed Recovery-Oriented Cognitive Therapy (CT-R), which provides concrete, actionable steps to promote recovery and resilience among individuals with serious mental health conditions. CT-R is beginning to change the way severe mental illness is conceptualized and treated. Initial research has supported this approach (Grant et al., 2012; Grant et al., 2017). Originally developed to treat schizophrenia, the principles of CT-R can be incorporated into CBT (J. Beck, 2020) and may be especially useful for individuals experiencing extensive behavioral, social, and physical health challenges. CT-R is highly collaborative, person-centered, and strengths-based, focusing on developing and strengthening positive beliefs of purpose, hope, efficacy, empowerment and belonging (and deemphasizing a focus on symptoms and negative beliefs). This approach has been implemented in a variety of inpatient, residential, and community settings, resulting in the reduction or elimination of controlling interventions such as seclusion, restraint, and as-needed medication, as well as reducing the length of hospital stays for individuals (Beck et al., 2020).

## The Future of Cognitive Therapy

Building on CBT’s demonstrated efficacy, one important continuing challenge for researchers and clinicians is to develop ways to deliver quality CBT treatment to the individuals who need it most. This involves both adapting treatment for diverse cultures and populations and creating effective and efficient treatment delivery models, including the expansion of digital and online methods of delivery and integrating CBT into primary care settings and public health clinics. It also entails robust and effective training programs for health and mental health professionals, peer specialists, care givers, teachers, and other groups. Dr. Aaron Beck has devoted his life to alleviating human suffering through his study and application of psychological principles. The CBT community looks forward to honoring his 70-year legacy by continuing to study and disseminate evidence-based CBT around the world.
